# A Public-Spirited Governor

**Published:** 1918-10-12

**Authors:** 


### A Public-Spirited Governor.

### A GREAT WORK AND ITS RECOGNITION.

THE Mayor of Leicester, Alderman Jonathan North, J.P., lately presided over a gathering of exceptional interest, when Mr. W. E. Hincks, M.B.E., J.P., was presented with a War Bond for £1,000 and an illuminated address in recognition of his great services to the town of Leicester.

For many years Mr. Hincks was secretary to the Local Charity Organisation Society. He was the administrator of the Prince of Wales' Fund and afterwards the chairman of the Local Naval and War Pensions Committee. His organisation was regarded as a model for other Centres, and his services were often taken advantage of by the Ministry of Pensions to advise other localities as to method and system; Glasgow and Cardiff were among the towns so visited. It was not, therefore, a great surprise locally that Mr. Hincks had been chosen by the Ministry of Pensions as their inspector for the North Midland area, a position which everyone felt he was eminently fitted to fill satisfactorily and creditably.

Mr. Hincks' public work for Leicester can be gauged by the fact that he has been for years a member of the Town Council, he was at one time chairman of the Libraries Committee, and for several years past has been, and still is, the chairman of the Watch Commitee. He is a Justice of the Peace for the borough, and his work for the Ministry of Pensions and for Leicester was recognised by His Majesty the King in the recent conferment of membership of the Order of the British Empire. Mr. Hincks is a member of the committee of the Leicester Saturday Hospital Society, of the board of governors of the Royal Infirmary, and a valuable member of many other local committees and societies. Grateful recognition was also made at the self-sacrifice of Mrs. Hincks, which had enabled her husband to devote his time to public work, in the presentation to her of a silver service suitably inscribed.

Mr. Hincks, in returning thanks for the gifts, looked forward to the time when a big scheme could be put into operation to ensure that no man, woman, or child should suffer material distress through the war.

